# “OPTIONS-DC”, a feasible discharge planning conference to expand infection treatment options for people with substance use disorder

**DOI:** 10.1186/s12879-021-06514-9

**Published:** 2021-08-09

**Authors:** Monica K. Sikka, Sara Gore, Taylor Vega, Luke Strnad, Jessica Gregg, Honora Englander

**Affiliations:** 1 Division of Infectious Diseases, Department of Medicine, Oregon Health and Science University, 3181 SW Sam Jackson Park Rd, L457, Portland, OR 97239-3098 USA; 2 School of Medicine, Oregon Health and Science University, Portland, OR USA; 3 Epidemiology Programs, School of Public Health, Oregon Health and Science University and Portland State University, Portland, OR USA; 4 Section of Addiction Medicine, Division of General Internal Medicine, Department of Medicine, Oregon Health and Science University, 3181 SW Sam Jackson Park Rd, BTE 119, Portland, OR 97239-3098 USA; 5 Division of Hospital Medicine, Department of Medicine, Oregon Health & Science University, Portland, OR USA

**Keywords:** Substance use disorder, OPAT, Harm reduction, Patient-centered care

## Abstract

**Background:**

Serious bacterial infections associated with substance use often result in long hospitalizations, premature discharges, and high costs. Out-of-hospital treatment options in people with substance use disorder (SUD) are often limited.

**Methods:**

We describe a novel multidisciplinary and interprofessional care conference, “OPTIONS-DC,” to identify treatment options agreeable to both patients and providers using the frameworks of harm reduction and patient-centered care. We retrospectively reviewed charts of patients who had an OPTIONS-DC between February 2018 and July 2019 and used content analysis to understand the conferences’ effects on antibiotic treatment options.

**Results:**

Fifty patients had an OPTIONS-DC during the study window. Forty-two (84%) had some intravenous (IV) substance use and 44 (88%) had an active substance use disorder. Participants’ primary substances included opioids (65%) or methamphetamines (28%). On average, conferences lasted 28 min. OPTIONS-DC providers recommended out-of-hospital antibiotic treatment options for 34 (68%) of patients. OPTIONS-DC recommended first line therapy of IV antibiotics for 35 (70%) patients, long-acting injectable antibiotics for 14 (28%), and oral therapy for 1 (2%). 35 (70%) patients that had an OPTIONS-DC completed an antibiotic course and 6 (12%) left the hospital prematurely. OPTIONS-DC expanded treatment options by exposing and contextualizing SUD, psychosocial risk and protective factors; incorporating patient preferences; and allowing providers to tailor antibiotic and SUD recommendations.

**Conclusions:**

OPTIONS-DC is a feasible intervention that allows providers to integrate principles of harm reduction and offer patient-centered choices among patients needing prolonged antibiotic treatment.

**Supplementary Information:**

The online version contains supplementary material available at 10.1186/s12879-021-06514-9.

## Background

Serious bacterial infections resulting from complications of substance use are rising amidst a substance use epidemic across the United States [1, 2]. Patients with substance-use associated infections commonly have long hospitalizations, high rates of leaving the hospital prematurely (“against medical advice" (AMA)) [3], and high costs associated with their care [4]. Infections such as endocarditis and osteomyelitis are commonly treated with many weeks of intravenous (IV) antibiotics [5, 6]. Yet for people with substance use disorders (SUD), treatment options are usually limited. Providers often deem an outpatient antibiotic plan unsafe due to concerns over risks of central venous access and active drug use [7]. Patients are commonly denied admission to skilled nursing facilities (SNF) [8] and SUD treatment settings are not equipped to manage antibiotic infusions [7]. Many patients do not or cannot stay in the hospital for weeks [9]. Preferences of patients with SUD are often disregarded or ignored and instead treatment plans reflect provider priorities [3]. This can lead to premature discharges, under-treated infections, and readmissions. Current care models often overlook this complex dynamic [10–13].

Our hospital has well-established infectious diseases (ID) and addiction medicine teams [14, 15], but lacked processes to prioritize patient preferences, optimize infection outcomes, and reduce harms of drug use among people needing long-term IV antibiotics. To address these gaps, we created a novel multidisciplinary and interprofessional care conference, “OPTIONS-DC,” to identify treatment options agreeable to both patients and providers using the frameworks of harm reduction and patient-centered care. In our model, harm reduction principles include not only mitigating the harms of substance use, but also compassion and respect for patient priorities regarding their infection treatment. The goal of this manuscript is to describe OPTIONS-DC and share ways OPTIONS-DC expanded treatment options and influenced provider decision-making.

## Methods

### Setting

We developed OPTIONS-DC at Oregon Health & Science University (OHSU), an urban academic medical center in Portland, Oregon. OHSU has a robust addiction consult service (ACS) that includes addiction medicine physicians, advanced practice providers, social workers (SW), and peer mentors with lived experience in recovery. Earlier work describes development and outcomes of our ACS [14–19]. We also have a comprehensive nurse care-coordinator managed outpatient parenteral antimicrobial therapy (OPAT) program comprised of a medical director, registered nurse (RN) navigators, and an ID trained pharmacist. The OPAT RNs navigate patients from hospital discharge to outpatient completion of their OPAT course. Before OPTIONS-DC, the ACS, ID, and OPAT teams commonly cared for the same patients but lacked standardized processes to identify post-hospital treatment options and support shared decision-making with patients. Home infusion, therapy at an infusion center, and long-acting injectable antibiotics were available for patients with SUD, but were not commonly supported or recommended by the ID team. A history of SUD alone was often considered to be unsafe for OPAT and individual factors were rarely considered. Communication and sharing of information between care teams was limited. Often, patients were required to complete long courses of IV antibiotics in-hospital, leading to patient and staff conflict, distress and poor outcomes [20].

### Conference development and description

As key stakeholders from OPAT, ACS, and ID, we developed OPTIONS-DC after unsuccessful efforts at providing integrated antibiotic infusion in residential treatment [7], ongoing patient and provider concerns that existing systems were failing patients, and having a series of patients leave the hospital before completing recommended antibiotic therapy. One patient wrote a letter to providers before leaving that made a searing impression. Their letter read:

“I have made my goals, concerns, and intentions as plain as I could from [out of] the gate. You don’t have to agree with them, or even fully understand them. That’s my job. Your job is to find the best means of healing my condition, without fairytale delusions.”

**Fig. 1:**
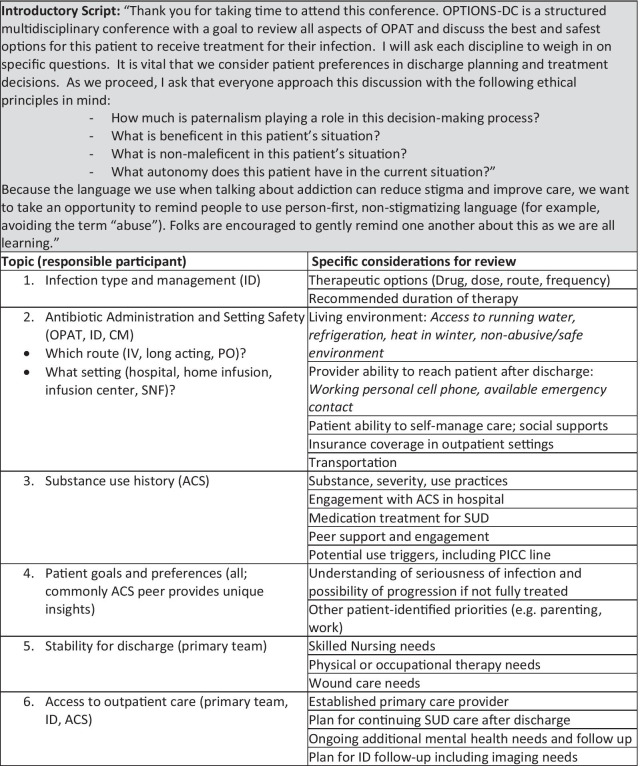
OPTIONS-DC Conference Format

OPAT, ACS, nursing, care management (CM), and hospital medicine teams convened to discuss our approach. There was consensus that patient care plans focused on provider—not patient—priorities and were generally stringent, failing to incorporate harm reduction principles. Over a series of meetings, we developed a standardized conference tool [21] to support providers to integrate patient preferences, identify and mitigate risks. Figure 1 displays a summary of the conference tool; we include the complete document in an Additional file 1: Appendix.

OPTIONS-DC participants include the primary hospital team, the ACS (medical provider, SW and peer), an ID physician, the OPAT pharmacist and RN, a hospital CM, and sometimes the bedside nurse. The patient was not included but their preferences were often relayed by the peer or ACS SW. An OPAT RN facilitates the conference, following the standardized format which begins with a script that clarifies the intent of the meeting, reminds participants of ethical principles in which to ground decisions (specifically patient autonomy, beneficence and non-maleficence) and reminds participants of the importance of non-stigmatizing language.

The standardized conference tool guides participants to review infection synopsis and treatment recommendations, substance use history including routes, frequency, use practices (e.g. access to clean syringes), engagement with ACS, addiction medication treatment (e.g. methadone, buprenorphine), peer support, peripherally inserted central catheter (PICC) safety as an outpatient, patient goals, other medical needs outside of the hospital, access to a primary care provider, insurance coverage, home environment, and access to phone and emergency contacts. After this guided review, providers discuss treatment options and alternatives. Then, one or more of the participants meets with the patient to discuss options elucidated at OPTION-DC and help make shared decisions around the ultimate treatment plan.

### Patient selection

Any member of the treatment team can request an OPTIONS-DC for patients with SUD needing prolonged IV antibiotics. The ACS administrative coordinator then schedules a meeting time.

### Data collection

The OPAT RNs who facilitate OPTIONS-DC prospectively maintained a database with patient demographics (age, gender (male/female), housing status (secure, insecure), access to working phone (yes/no), substance use information (primary (free text), route (intravenous, intramuscular, inhaled, oral), active substance within last 90 days (yes/no)). We also documented primary infection site (bone/joint, endovascular, intra-abdominal, bacteremia, other/multiple), pre-conference and post-conference antibiotic recommendations including choice, route, and setting, plan for medication for opioid use disorder (MOUD) if indicated, conference participants, and duration.

After obtaining approval from the OHSU Institutional Review Board, we retrospectively reviewed charts of all patients who had an OPTIONS-DC between February 2018 and July 2019. We abstracted ultimate antibiotic administration location and route, discharge location, hospital length of stay, number of out-of-hospital antibiotic days, premature discharges, and whether the patient completed an antibiotic course. Four members of the research team (MS, SG, LS, HE) participated as clinicians in some conferences and provided additional insights on specific cases.

### Data analysis

We used descriptive statistics to report patient demographics, conference recommendations and outcomes. We performed content analysis [22] of the chart notes focusing on documentation of the OPTIONS-DC conference. We created summaries of four main conference content areas: changes to antibiotic plan, changes to SUD plan, risk and protective factors, and consideration of patient preferences. Three coders (S.G., M.S., T.V) independently coded chart notes and met in dyads to reconcile codes. The full team met periodically to identify patterns in which OPTIONS-DC affected patient treatment options. We ended content analysis after reaching theme saturation and identified key examples derived from the summaries.

## Results

Fifty unique patients had an OPTIONS-DC during the study window. Patients’ mean age was 40, 42 (84%) reported any injection substance use, and 26 (52%) had unstable housing. We describe patient demographics in Table 1. On average, conferences occurred on hospital day 14 and lasted 28 min. OPTIONS-DC providers recommended IV antibiotics for 35 (70%) of patients, long-acting injectable antibiotics for 14 (28%), and oral therapy for 1 (2%). The most common locations recommended for antibiotic completion were home for 19 (38%) patients, in hospital for 15 (30%), or at SNF for 10 (20%). Thirty-five (70%) patients completed an antibiotic course recommended by OPTIONS-DC. Twenty (40%) patients ultimately discharged home to complete antibiotics. Of those, 4 received home infusions, 4 went to an infusion center, 9 received a long-acting injectable antibiotic, and 3 received an oral antibiotic. Six patients (12%) left the hospital prematurely, before completing recommended therapy. Mean hospital length of stay was 28 days with mean out-of-hospital antibiotic days of 12. Table 2 summarizes conference details and clinical outcomes.

**Table 1:** Patient demographics

Demographics	Patients (N = 50)
Mean age, y	40
Female, n (%)	22 (44)
Insecure housing, n (%)	26 (52)
Working phone, n (%)	34 (68)
Substance use	
Primary substance use, n (%)	
Opioids	34 (68)
Methamphetamines	14 (28)
Both opioids and methamphetamines	2 (4)
Intravenous use, n (%)	42 (84)
Active substance use disorder (SUD), n (%)	44 (88)
Initiation of medication for opioid use disorder (MOUD) during hospitalization, n (%)	32 (64)
Primary infection site	
Bone/joint, n (%)	24 (48)
Endovascular, n (%)	10 (20)
Intra-abdominal, n (%)	3 (6)
Bacteremia, n (%)	7 (14)
Other/multiple, n (%)	6 (12)

**Table 2:** Conference details and clinical outcomes

OPTIONS-DC conference details	N = 50
Mean conference duration (minutes)	28
All teams^a^ present, n (%)	39 (78)
Antibiotic route recommended after OPTIONS-DC, n(%)	
Intravenous	35 (70)
PO	1 (2)
Long-acting injectable	14 (28)
Planned location to complete antibiotics, n (%)	
In Hospital	16(32)
Out of Hospital	34 (68)
Home	19 (38)
Skilled nursing facility	10 (20)
Shelter	2 (4)
Residential treatment	2 (4)
Jail	1 (2)
Clinical outcomes	
Discharged on intravenous antibiotics, n (%)	16 (32)
Discharged home, n (%)	20 (40)
Home Infusion, n (%)	4 (20)
Infusion center, n (%)	4 (20)
Long-acting injectable, n (%)	9 (45)
Oral antibiotics, n (%)	3 (15)
Left hospital prematurely, n (%)	6 (12)
Did not complete therapy or lost to follow-up, n (%)	8 (16)
Died in hospital, n (%)	1 (2)
Completed an OPTIONS-DC recommended therapy, n (%)	35 (70)
Mean hospital length of stay, days (SD)	28 (13.4)
Mean out-of-hospital antibiotic, days (SD)	12 (12.8)
Total out-of-hospital antibiotic days, days	603

^a^Addiction consult service (ACS), Outpatient parenteral antimicrobial therapy (OPAT) team, Infectious Diseases (ID), and primary team

Content analysis supported that OPTIONS-DC expanded infection and addiction treatment options by: (1) exposing and contextualizing SUD, psychosocial risk and protective factors; (2) incorporating patient preferences into care plans; and (3) creating space for providers to weigh risks and benefits to tailor antibiotic and SUD recommendations. Using the following case examples, we describe how the conferences worked.

### Exposing and contextualizing SUD, psychosocial risk and protective factors

The OPTIONS-DC format supported a systematic way to discuss patient risk and protective factors, specifically patients’ living environment, substance use, ability to self-manage medical care, and access to post-hospital follow-up. This information sometimes led providers to modify the discharge plan to recommend completion of antibiotics in an outpatient setting.

#### Living environment

For example, one patient with history of IV methamphetamine use and culture-negative septic arthritis lived in a mobile home that lacked running water, thus precluding home infusion. OPTIONS-DC centered treatment options around the patient’s strong preference to return home. Providers weighed the benefit of offering a long-acting injectable antibiotic at an infusion center as a feasible alternative to completing empiric IV antibiotics in-hospital or at a SNF.

Another patient with history of inhaled methamphetamine use and aortic valve endocarditis felt that returning to their hometown was a strong trigger for return to substance use; however, insurance would only cover 20 days at a SNF and providers worried that no SNF would accept the patient given their SUD. The patient’s partner lived in a different town and was a sober support. Holding these varied considerations, conference providers supported home infusion at the partner’s home.

#### Substance use

Often, conferences revealed risk or protective factors that were either unrecognized or difficult to contextualize. For example, one patient with methamphetamine use was admitted with methicillin-resistant *Staphylococcus aureus* (MRSA) bacteremia and a spinal epidural abscess. During OPTIONS-DC, the ACS SW shared that the patient had a mild use disorder, was deeply religious, and had past periods of prolonged abstinence during Lent, which corresponded with the recommended treatment course. Given these protective factors and lack of injection drug use history, the team offered the option of completing daily daptomycin via home infusion.

Sometimes, OPTIONS-DC led providers to recommend antibiotic completion in the hospital. One patient with lumbar osteomyelitis and psoas abscesses, who started methadone during hospitalization, worried that completing IV antibiotics at her home with a roommate with active use would be too triggering. Given the serious infection, not-yet blocking dose of methadone, and patient preference, OPTIONS-DC recommended completing antibiotics in-hospital.

#### Patient’s ability to self-manage care after discharge

In one instance, OPTIONS-DC discussed a patient with a history of inhaled methamphetamine use and pelvic osteomyelitis whose partner had recently completed an OPAT course. Both she and her partner felt confident in their ability to manage antibiotics via home infusion. Based on the assessments of risks and benefits, OPTIONS-DC offered her this option. In contrast, for a patient with high-grade MRSA bacteremia, the ACS SW outlined a history of leaving the hospital prematurely, documented memory problems, and poor adherence with follow-up appointments. This led to concern for adherence with home infusions and compliance with treatment at an infusion center; instead, providers recommended a long-acting injectable antibiotic on discharge to ensure completion of therapy.

#### Insurance coverage and post-hospital follow-up

Guided mostly by hospital CM, OPTIONS-DC participants systematically reviewed insurance coverage for transportation, outpatient infusions, SNF care, and outpatient follow-up. This assured the feasibility of frequently complex discharge plans. For one patient, OPTIONS-DC clarified that with Medicare alone, there was no coverage for medical transportation making daily antibiotics at an infusion center and daily methadone clinic visits unrealistic. As such, the patient completed antibiotics in-hospital.

### Incorporating patient preferences into care plans

OPTIONS-DC allowed providers to tailor infection treatment by balancing guideline-recommended therapy with patient preferences, feasibility, and effectiveness for the individual patient. Though the patient was not present at OPTIONS-DC, their priorities and wishes were shared with providers by the peer or ACS SW.

One woman with a spinal epidural abscess and MRSA endocarditis wanted to discharge to her daughter’s home, but was reluctant to have a PICC, fearful it would trigger return to use. OPTIONS-DC created a space to weigh infection risks with the patient’s preferences and concerns regarding SUD and resulted in the option of oral antibiotics at her daughter’s home as an alternative to possible premature discharge.

There were many patients reluctant to complete extended antibiotic courses in-hospital. OPTIONS-DC discussions focused on supporting patients given often limited outpatient options, and developing contingency antibiotic plans should they choose to leave the hospital. For example, one woman admitted with *Staphylococcus aureus* tricuspid valve endocarditis developed metastatic complications during hospitalization requiring extension of her antibiotic course. OPTIONS-DC providers recommended completing therapy in-hospital given her unstable housing, her concern regarding injecting into a PICC, lack of SNF availability, and the opportunity for ongoing ACS involvement. OPTIONS-DC outlined a plan to transition to an oral agent if she were no longer able to tolerate hospitalization, which occurred with 3 weeks of therapy remaining. While not ideal, contingency plans outlined during OPTIONS-DC led to less chaos when patient preferences changed abruptly and allowed for some treatment instead of potentially none.

### Creating space for providers to weigh risks and benefits of treatment options and tailor antibiotic and SUD recommendations

In each conference, OPTIONS-DC facilitated group decision-making and allowed providers to weigh risks and benefits of treatment options. For a patient taking methadone admitted with *Staphylococcus aureus* meningitis and endocarditis, drug-drug interactions between methadone and the combination of levofloxacin with rifampin precluded the use of oral antibiotics for the last four weeks of therapy. However, in OPTIONS-DC, it surfaced that the patient could transition from methadone to buprenorphine to allow for use of the recommended oral antibiotics. Alone, one discipline would have been unable to tailor a treatment plan that met the needs of the whole person; together, a plan was formulated that met the patient’s goals and optimized likelihood of cure.

Another example was weighing the risks and benefits of a PICC in a patient with a history of daily IV heroin use and self-directed discharges from SNFs. This patient would have previously been considered too high risk for a PICC as an outpatient. However, at OPTIONS-DC, many protective factors were revealed, including engagement with ACS and motivation to treat his infection. Per the patient’s preference, he was discharged with a PICC for daily infusions at an infusion center, referred for housing through a recuperative care program, and linked to outpatient SUD treatment. This complex scenario would have been difficult for any team to individually navigate, but OPTIONS-DC was a forum to tailor SUD and antibiotic therapy so no one provider had to shoulder the risks associated with the ultimate therapeutic decision.

## Conference limitations

Sometimes OPTIONS-DC was unsuccessful in identifying treatment choices that aligned patient and provider goals. Medical complexity, lack of social supports, inability to secure a safe or stable discharge location, and lack of reliable transportation or modes of communication are some risk factors that may have outweighed the potential benefits of honoring patients’ goals. For a patient with a complicated pelvic infection requiring interval reimaging who wanted to return home, the medical teams felt her home environment was unsafe for home infusion and her ongoing medical needs made an inpatient plan the safest choice. She ultimately left the hospital prematurely.

Further, sometimes barriers such as SNF denials, insurance limitations, and dispensing outpatient methadone prevented execution of OPTIONS-DC recommendations. For one patient with a complicated bacteremia who was living in his car prior to admission, OPTIONS-DC recommended completion of IV antibiotics at a SNF. When coordinating this plan, no SNF would accept him because of his SUD history. He ultimately completed antibiotics in-hospital.

In almost a quarter of the conferences, not all team members were present which sometimes resulted in incomplete knowledge of potential discharge options and delay in developing a discharge plan. Finally, because conferences often took place well into a hospital course, patients who left prematurely prior to coordination of an OPTIONS-DC may have been excluded.

## Discussion

A structured, interprofessional provider conference is feasible and has the potential to improve patient care by exposing and contextualizing SUD, psychosocial risk and protective factors; incorporating patient preferences into care plans; and allowing providers to tailor antibiotic and SUD recommendations to whole-person, real-life circumstances. In real-world settings, first-line antibiotic treatments may fail patients [23], as guidelines may not consider various patient-level factors, including SUD, housing insecurity, and patient preferences [5, 6]. Whereas previously many patients, particularly those with endocarditis and osteomyelitis, were offered only in-hospital antibiotic treatment resulting in longer than expected lengths of stay [14], we found that in patients who had an OPTIONS-DC, over two-thirds completed antibiotics outside of the hospital, including 40% who discharged home.

Our findings build on literature describing care models for patients with SUD needing prolonged antibiotics. Earlier work describes risk assessment tools [24] and eligibility criteria [25] for IV antibiotics outside of the hospital. Our work augments this, providing a structured way for providers to help one another understand and weigh possible risks and benefits to tailor care plans towards patient-centered goals. Our work also builds on literature describing models [7, 26] that combine intensive MOUD and OPAT in outpatient or residential addiction treatment settings. Unlike potentially more complex efforts to redesign care systems, OPTIONS-DC is simple and serves largely as a provider decision support tool that emphasizes harm reduction and patient priorities. Finally, our findings add to literature examining optimal antibiotic routes for treating serious infections [27, 28]. While earlier studies question the need for prolonged courses of IV antibiotics for endocarditis or osteomyelitis [27, 28], they excluded or had few patients with SUD—patients who might struggle with adherence and to whom healthcare providers struggle to listen to as partners in clinical decision-making. Our experience provides a framework to including patient voices.

Our study has several limitations. First, this is a descriptive study without a comparison group. Our model is used at a single academic medical center with ID, OPAT, and an ACS and thus may not be generalizable to settings without this expertise. Further, while we describe treatment planning processes, we do not describe long term outcomes beyond completion of recommended antibiotic courses. Finally, our methods do not assess patient or provider perspectives on OPTIONS-DC and antibiotic decision-making.

Our model has important implications for patient care, health systems, and future research. OPTIONS-DC is a simple tool that can be adapted to other scenarios. For example, our ACS is adapting the OPTIONS-DC structure to develop a shared conference with palliative care to support care planning and end-of-life decision-making. Our ID, cardiology, and cardiac surgery groups are adapting it for decision-making about cardiac valve replacement in endocarditis. Our experiences could also inform complex interdisciplinary provider discussions around advanced heart failure therapies and organ transplantation. The successes of OPTIONS-DC demonstrates how hospital providers can integrate harm-reduction principles into care, and highlights the value of addiction medicine and ID cross-training and collaboration [29]. Finally, our model is built on strong consideration of ethical principles and shared decision-making which emphasizes the need to increase trust and collaboration between providers and patients when developing care plans.

Our experience highlights important implications for health systems. Traditionally hospitals are hierarchical and inflexible systems, and structures within these settings do not support providers to adapt care plans to meet patients’ needs and goals. Our experience shows that by superimposing collaborative structures into complex decision-making, we can change this.

Future research with patient and provider interviews could provide understanding of mutual mistrust [30, 31] amongst patients and providers and how to build systems to overcome barriers that contribute to this mistrust. Future work should also explore in-depth patient and provider perceptions as well as long-term clinical outcomes.

## Conclusion

As infectious complications of SUD continue to rise in the US, OPTIONS-DC is a feasible tool that offers alternatives to long hospitalizations. Our experience is adaptable to other hospital settings with addiction medicine and ID care, and can support better contextualizing and framing of patient-level risk and protective factors, to align care plans and patient priorities.

## Supplementary Information

**Additional file 1.** OPTIONS-Dc meeting tool.

## Data Availability

The data used/analyzed for this study are available from the corresponding author on reasonable request.
