# Supplementary material for: “OPTIONS-DC”, a feasible discharge planning conference to expand infection treatment options for people with substance use disorder
Source: BMC Infect Dis. 2021 Aug 9;21:772. doi: 10.1186/s12879-021-06514-9 (PMC8351414; doi:10.1186/s12879-021-06514-9)
Supplement: Supplementary file 1 — Additional file 1. OPTIONS-Dc meeting tool. [file 12879_2021_6514_MOESM1_ESM.docx]

Appendix: OPTIONS-DC Meeting Tool:

Facilitated and documented in Epic by OPAT RN

**Introduction to Care Conference:**

**“Thank you for taking time to attend this care conference. This is a structured multidisciplinary care conference with a goal to review all aspects of OPAT and discuss the best and safest options for this patient to receive treatment for their infection. I will ask each discipline present to weigh in on specific questions/concerns. It is vital that we consider patient preferences in discharge planning and treatment decisions. As we proceed, I ask that everyone approach this discussion with the following ethical principles in mind:**

- **How much is paternalism playing a role in this decision-making process?**
- **What is beneficent in this patient’s situation?**
- **What is non-maleficent in this patient’s situation?**
- **What autonomy does this pt have in the current situation?”**

**Because the language we use when talking about addiction can reduce stigma and improve care, we want to take an opportunity to remind people to use person-first, non-stigmatizing language (for example, avoiding the term “abuse”). Folks are encouraged to gently remind one another about this as we are all learning.”**

Start time: ***, Stop time: ***

Team members involved:

Primary team: ***

IMPACT team: ***

Case Manager: ***

ID team: ***

OPAT Team: ***

| **SYSTEMATIC REVIEW OF NEEDS, RISK and PROTECTIVE FACTORS** | |
| --- | --- |
| ID synopsis/recs with duration, antibiotic, dosing | *** |
| Substance use history: frequency, last use, delivery method | *** |
| Who triggered care conference?  Reason for care conference/ problem identified? | *** |
| Patient’s goal/perspective: | *** |
| SW PICC safety in community assessment: | *** |
| Is patient medically stable for discharge? (primary team) | *** |
| Does patient have skilled needs ie  PT/OT/ADL/wound care needs? (primary team) | *** |
| Insurance coverage options per CM | *** |
| Does the patient have a working, personal cell phone?  How do we know it is working? | *** |
| Updated emergency contacts and addresses | *** |
| Is home environment safe? (running water, refrigeration, heat in winter, non-abusive/safe environment) | *** |
| Transportation WITH funds for transportation AND willingness to travel. (Applies to all potential dc settings) | *** |
| Previous OPAT course history (if applicable) | *** |
| Receiving treatment for mental health condition post dc if applicable?  Are they on medications? Are they able to obtain medication? | *** |
| Receiving addiction treatment post dc? Medications, counseling, peer support, etc. | *** |
| Does pt have a PCP? Is f/u appt made? | *** |
| Has there been transparency from all teams regarding the seriousness of the infection and disease progression if infection is not treated/not treated optimally.  Has there been transparency about potential health risks with PICC line or misused PICC line (i.e., injecting into PICC line, injecting into other veins while having PICC line in, getting dressing wet, lack of dressing change q7 days or prn, line pulled out/line pushed in, DVT, rash/irritation)?  Is PICC line a trigger for substance use in this patient? | *** |
| **DISCUSSION AND RECOMMENDATIONS** |  |
| Options discussed for treating infection from ID standpoint:   1. Most ideal antibiotic option and setting based on patient’s infection and potential for antibiotic completion. 2. Alternate option: 3. Alternate option:   Likelihood of antibiotic success for treatment of infection. | See above |
| Who will discuss options/team perspective with patient? | *** |
| Infectious Diseases contact person/team if questions arise after this date: | *** |

Paternalism: A philosophy that certain health decisions are best left in the hands of those providing healthcare.

Beneficence: is action that is done for the benefit of others. Beneficent actions can be taken to help prevent or remove harms or to simply improve the situation of others.

Non-maleficence: to “do no harm.” A principle of bioethics that asserts an obligation not to inflict harm intentionally.

Autonomy: the right of competent adults to make informed decisions about their own medical care.
